# Cultural modification of neuropsychiatric assessment: complexities to consider

**DOI:** 10.1192/bjo.2022.33

**Published:** 2022-03-15

**Authors:** Sandila Tanveer, Matthew J. Croucher, Richard Porter

**Affiliations:** Department of Psychological Medicine, University of Otago, Christchurch, New Zealand; Department of Psychological Medicine, University of Otago, Christchurch, New Zealand; and Canterbury District Health Board, Christchurch, New Zealand; Department of Psychological Medicine, University of Otago, Christchurch, New Zealand; and Canterbury District Health Board, Christchurch, New Zealand

**Keywords:** Dementia, low- and middle-income countries, psychological testing, cognitive neuroscience, depressive disorders

## Abstract

Cognitive screening tests are culture bound and have been shown to perform differently depending on the culture, even with adequate translation. Khan et al examine in detail ways in which the Montreal Cognitive Assessment (MoCA) has been modified for different languages and cultures and produce a systematic guide for future modifications. However, questions arise regarding the availability of the MoCA. Other important issues in the transcultural use and modification of neuropsychiatric tests include providing a culturally safe context for testing, understanding the cultural context in which screening takes place and assessing other neuropsychiatric conditions, which may manifest differently in different cultural contexts and which affect cognition.

Most assessments of neuropsychiatric function are written in English and are premised on the cultural norms of North American and European populations. Indeed, they are based on these cultures’ conceptualisations of neuropsychiatric disorders, which have dominated neuropsychiatric research. This issue extends to instruments assessing cognitive function. Short assessments that can provide a feasible and reasonably accurate screen for significant cognitive decline of various aetiologies are at a premium, but may not perform as well in languages and cultures beyond that in which they were developed.^1^ Simply translating these measures into other languages is unlikely to maintain their usefulness cross-culturally.

In their excellent review and their development of guidelines for the adaptation of the Montreal Cognitive Assessment (MoCA^©^), Khan et al^2^ examined 52 publications reporting on language and cultural adaptations of the MoCA that attempted to maintain its integrity. Problems that were overcome included the picture of a rhinoceros not being readily recognisable in certain countries or the memory-testing words being modified to words with a similar semantic frequency in the language being used. However, many of the adaptations had often been conducted in a somewhat *ad hoc* manner. By pulling together the issues that have been addressed, Khan et al arrived at a systematic guide for the adaptation of the MoCA for any culture and language, which has the potential to be of great utility as a template for adapting the MoCA and other cognitive tests in future. In this area, the paper goes further than another recent review^3^ on the same topic.

Of course, this excellent review can only address certain aspects of this complex area and there are several other issues that will need further detailed research. Below we consider some of these issues in the hope of signalling areas for future research.

## Availability of cognitive tests

Particularly in low- and middle-income countries (LMICs) an important aspect of the feasibility of cognitive screening tools, and indeed more detailed cognitive tests, is cost. Being able to copy multiple single sheets of a screening tool at low cost and to administer this with minimal training in a reasonably reliable way is vital. However, in 2010 the licensee of the exclusive right to publish and distribute the Mini-Mental State Examination (MMSE^©^) began to assert their rights. Subsequently, the owner of the MoCA asserted their rights from 2019, stating their wish to limit access to users who maintain individual accreditation to use the test via a fee-for-service in-house online training platform every 2 years. Unlike the case of the MMSE, we are not aware of any legal attempt to date to assert the MoCA's copyright conditions. The option of negotiating group access agreements is possible for the MoCA at least, potentially nationally, but these developments have made the use of the MMSE and MoCA unfeasible for publicly funded health services in most LMICs, if not most countries. Adaptations of the English MMSE into other cultural and linguistic settings are explicitly covered by its licence agreement and strongly implied for the MoCA. Were the developers of such adaptations not allowed to continue to use them free of charge within their health services then this would result in their having wasted vast amounts of time and resources. We sincerely hope that the originators of the MoCA address this issue in a pragmatic way. We also hope that the excellent work of Khan et al can be built on by allowing future adaptations in LMICs without the issue of copyright making this impractical. We note also the excellent previous work^4^ of Khan et al's group on the Addenbrooke's Cognitive Examination III (ACE-III^©^), a scale whose copyright owner has explicitly stated that ‘the guiding principle of the ACE-III is that it is free for [clinical and research] users’,^5^ and hope that use and adaptation of this scale and its related instrument, the Mini-ACE^©^, can continue without future copyright issues.

## Validation of brief cognitive screening tests

As Khan et al^2^ point out, the ultimate validation of adaptations of scales is their use in conjunction with more definitive diagnostic measures. The more definitive diagnosis of dementia against which the sensitivity and specificity of the MoCA have been tested is based on the performance of other cognitive tests or on comprehensive clinical assessment by experts. Both validation methods may be challenging to accurately adapt to settings culturally and linguistically different from the populations in which the original instruments were validated. These problems are mitigated to an extent by norms for cognitive tests having been determined in the population in question and by the use of internationally standardised diagnostic criteria. However, there are risks to be overcome at every stage of this process.

## Cultural context of administration

An additional problem is the context of administration of the test. For example, in the context of working with Māori, the indigenous people of New Zealand, it has been suggested that cognitive testing may be interpreted as a challenge or *wero,* which would seriously impair engagement with the testing process, highlighting the importance of setting up a culturally safe context for testing. In general, it has been demonstrated that people being assessed by a clinician of the same culture/ethnicity as them, and with a culturally appropriate^6,7^ process of engagement such as formal greetings or a prayer, increases engagement in the testing process and may improve performance^8^ and therefore reliability. A system in which a culturally appropriate test is used but in which the correct cultural context is neglected may risk lack of engagement and false positives.

## Should screening tests be developed *de novo*?

The question should be asked whether, at least in some cultures, neuropsychological instruments should be developed based on an understanding of that culture and on fundamental neuropsychological principles, rather than adapting an existing test developed in a different culture. This is indeed being attempted in New Zealand^9^ with the development of a kaupapa Māori instrument, the MANA. Of course, such tests still have to be validated against a broader cognitive battery and/or expert clinical assessment which may be less culture specific, and the approach is much more resource intensive than adapting a current test that already has extensive validation data. However, where resources exist the *de novo* approach has many potential advantages. An alternative approach is to develop a test that appears to perform across the range of cultures existing in a particular region. An example is the Rowland Universal Dementia Assessment Scale, which aims to be truly multicultural^10^ and therefore usable broadly in a multicultural society.

## Screening for other neuropsychiatric conditions

One of the limitations of dementia screening is that screening tests on their own do not recognise the impact of other mental health conditions. Depression, for example, is common in older individuals, particularly those who may be suspected of having a cognitive impairment, and may explain some or all of the impairments detected on cognitive screening instruments. Scales have been designed to screen for depression, but these are also likely to be subject to cultural differences and potentially need to be modified or have their cut-off points changed rather than simply being translated. For example, a recent study in this journal has illustrated important differences in symptom profiles in depression across a number of cultural groups, as expressed in the Beck Depression Inventory.^11^

## Fitting screening to the context

The detection of mild cognitive impairment can allow early appropriate assessment and management, such as the ‘prescription’ of strategies promoting brain health and setting up appropriate monitoring. However, the development of appropriate screening tools may be premature in areas of the world where resources do not exist to provide such treatment. Indeed, in some cultures, the approach to early dementia may be very different, with tolerance for or even an expectation of forgetfulness in the elderly, without the stigma of pathologisation or the immediate resort to medical treatments that may be seen in Western cultures (see for example Dudley et al^12^). Aggressive screening in this context may not be culturally appropriate.

## Conclusions

The article by Khan et al^2^ addresses a complex and difficult dilemma. Culture has important and pervasive effects on the testing and assessment of neuropsychiatric disease. Western literature has developed a suite of neuropsychological instruments which are frequently, but not always correctly, used in culturally and linguistically diverse settings. The dilemma exists regarding whether to recreate instrument development research within the alternate culture and language settings or to adapt existing measures. Khan et al provide excellent guidelines regarding how to embark on adaptation in the case of the MoCA, which may also prove useful for the adaptation of other tests, such as the Mini-ACE and ACE-III. The way in which any test is administered is also important to engagement, performance and ultimately reliability; and screening for other mental health problems also needs to be considered. Finally, much excellent work such as Khan et al's will be wasted if copyright issues and attendant costs continue to threaten the accessibility of modified screening tests.

## Data Availability

Data availability is not applicable to this article as no new data were created or analysed in its preparation.
